# Intention-to-treat and transparency of related practices in randomized, controlled trials of anti-infectives

**DOI:** 10.1186/s12874-016-0215-2

**Published:** 2016-08-24

**Authors:** Robert D. Beckett, Kathryn C. Loeser, Kathryn R. Bowman, Trent G. Towne

**Affiliations:** 1 Manchester University College of Pharmacy, Natural, and Health Sciences, 10627 Diebold Road, Fort Wayne, IN 46845 USA; 2 Columbus Regional Health, 2400 17th Street, Columbus, IN 47201 USA; 3 CVS Pharmacy, 1221 W Monroe Street, Decatur, IN 46733 USA

**Keywords:** Intention-to-treat, Per protocol, Anti-infectives, Clinical trial evaluation

## Abstract

**Background:**

Intention-to-treat (ITT) analysis is commonly recommended for use, due to its benefits on external validity, in randomized, controlled trials (RCTs). No published reports describe how ITT analysis, as well as alternative approaches, are used in anti-infective RCTs. The purpose of this study is to describe the extent to which ITT analysis and alternative data approaches are used, the practices used to handle missing subject data, and whether non-inferiority trials present both ITT and per protocol (PP) analyses. Results of this analysis will help guide end users of infectious diseases primary drug literature.

**Methods:**

A cross-sectional study of RCTs of anti-infectives published from January 1, 2013 through December 31, 2014 was conducted. A PubMed search identified relevant articles published in five specialty infectious diseases journals and four general medical journals. Each article was reviewed by two independent investigators with discrepancies resolved by consensus. Descriptive statistics were used to quantify results.

**Results:**

One hundred four articles met study criteria. The most common medication classes represented in the RCTs were hepatitis C antivirals (26 %), antibacterials (25 %), and antiretrovirals (21 %). Thirty studies (29 %) were non-inferiority trials. Most studies (77 %) described use of ITT or modified ITT (mITT) in their methods. Of the ITT and mITT studies, most (73 %) did not describe practices used to handle missing data. Most (97 %) non-inferiority trials described use of ITT, mITT, or both; however, only 15 (50 %) also described use of PP.

**Conclusions:**

RCTs of anti-infectives commonly employ ITT and mITT. Most do not describe how missing data were addressed. Non-inferiority trials of anti-infectives do not consistently employ both ITT and PP populations.

**Electronic supplementary material:**

The online version of this article (doi:10.1186/s12874-016-0215-2) contains supplementary material, which is available to authorized users.

## Background

As consumers of primary literature, medical professionals must become proficient in interpreting and evaluating published studies in order to determine applicability of results toward solving patient care problems. Similarly, investigators face many critical decisions when designing and conducting randomized, controlled trials (RCTs). One such decision is determining which study population to analyze. The three most commonly analyzed populations in RCTs are intention-to-treat (ITT), modified ITT (mITT), and per protocol (PP) [1, 2]. See Table 1 for definitions of these populations.

**Table 1:** Definitions of Data Analysis Populations^a^

Population	Definition^c^
ITT	All randomized patients are analyzed, regardless of whether each patient completed the trial, or even took a dose of study medication.^b^
mITT	All patients randomized patients are analyzed, if they fit a predefined modification to the ITT population. A common modification is including all patients who were randomized and also received at least one treatment dose.
PP	Only the patients who completed treatment according to the planned protocol are analyzed.

^a^ *ITT* intention-to-treat, *mITT* modified ITT, *PP* per protocol

^b^ITT may also be defined with the added caveat that there are no missing measurements; however, this standard was not applied in this investigation

^c^[2]

Analysis using ITT is generally preferred, and most often used, by RCTs [1, 2]. ITT analysis includes all patients regardless of adherence to study protocol and attrition, maximizing external validity, and more closely mirroring circumstances encountered in actual practice. This yields estimates of treatment effect that are more conservative compared to a PP analysis, and decreases risk for Type I statistical error [3, 13]. This approach also allows authors to preserve sample size [1–3]. However, a true ITT may include patients who have never received any study medication, regardless of treatment arm, which could be problematic, especially when evaluating non-inferiority. A mITT analysis may be preferred by investigators in order to balance the improved external validity obtained with ITT with the improved internal validity (i.e., assurance that study results accurately reflect the experiences of study subjects) associated with PP. For example, a mITT that only includes patients who received at least one dose of study medication is often employed in assessing differences in adverse drug events between treatment groups because it could be considered inappropriate to attribute an adverse drug event to a medication never received by the patient. Additional qualifiers such as the “microbiological” mITT, are sometimes added in the case of infectious diseases literature. Depending of the specific interventions and clinical outcomes being measured, an analysis of this depth may or may not be pertinent to assessment of the study’s impact on clinical decision making.

By including all patients regardless of violations or attrition, ITT tends to make the two treatment groups more similar [7]. For this reason, it has been proposed to be less appropriate in non-inferiority trials, where the goal of the investigation is to establish whether the intervention is “just as good” as the standard of care. In a non-inferiority trial, the null hypothesis is that the intervention is inferior to the standard, and an ITT population may be less conservative [4]. However, if an intervention is to be managed within a hospital where violations may be low due to improved adherence, ITT and PP findings should be similar. Regardless, both ITT and PP are recommended for presentation of results of non-inferiority trials, and the reader should be directed toward the more conservative findings.

When ITT and mITT are used, investigators must determine how to account for missing data due to attrition. Common approaches include using the last observation carried forward (LOCF) or assuming treatment failure when data are missing [2, 4]. The most ideal or preferred method has not been established, and investigators should consider conducting sensitivity analysis to investigate potential differences among methods. Regardless of which method is used, authors should describe the details of how they accounted for missing outcome data, as the approach taken could have substantial impact on the estimated effect size determined in the study.

CONSORT, the Consolidated Standards of Reporting Trials, provides standards for reporting clinical trials [1]. The CONSORT 2010 Statement recommends that authors who are reporting on clinical trials include “a clear description of exactly who was included in each analysis” regardless of which population is used. It is important for authors to provide details about which patients are included in the analysis so that readers have the information they need to interpret the results of the study. Furthermore it is important that the reported study populations in the methods section of the manuscript are consistent with what is utilized in the results and discussion section of the document. Failure to do so may lead to a misrepresentation of the true outcomes of a study.

Few previous studies describe whether published RCTs are meeting the CONSORT recommendation of clearly defining the population that was analyzed; however, available studies suggest significant gaps, particularly in describing how missing data are handled [5, 6]. In the absence of consistently applied principles, medical professionals are left to compare trials of similar drugs under potentially very different conditions. Similar data have not yet been described in a sample of RCTs of anti-infectives, a group of studies associated with high dropout rates, small sample size, and a high rate of non-inferiority trials [7]. Each of these issues could pose challenges related to use of ITT, mITT, and PP analyses.

In order to better understand use of ITT and related methods in published RCTs of anti-infectives, and how current practice benchmarks to best practices, the objectives of this study were to:

1. Describe the extent to which ITT analysis and alternative approaches to data analysis are used.
2. Describe practices used to handle missing data due to attrition.
3. Determine whether non-inferiority trials present both ITT and PP analyses.

## Methods

This was a cross-sectional study of RCTs of anti-infectives published from January 1, 2013 through December 31, 2014. Investigators conducted a preliminary literature search that identified a two-year search timeframe would likely identify approximately 100 RCTs meeting study criteria. A goal anticipated sample of 100 RCTs was selected based on prior similar studies [6, 8].

In order to be included, RCTs had to be published in one of nine medical journals: five specialty infectious diseases journals (i.e., *Antimicrobial Agents and Chemotherapy*, *Clinical Infectious Diseases*, *Journal of Antimicrobial Chemotherapy*, *Journal of Infectious Diseases*, *Lancet Infectious Diseases*) and four general medicine journals known to publish impactful RCTs of anti-infectives (i.e., *British Medical Journal*, *Journal of the American Medical Association*, *Lancet*, and *New England Journal of Medicine*). These journals were selected based on a prior study suggesting that they typically publish the most impactful RCTs of anti-infectives; they also represent the highest impact journals in their respective disciplines [9]. Impact factors ranged from 4.476 (*Antimicrobial Agents and Chemotherapy*) to 21.372 (*Lancet Infectious Diseases*) for infectious diseases journals and 17.445 (*British Medical Journal*) to 59.558 (*New England Journal of Medicine*) for general medicine journals [10–13]. RCTs that had a non-efficacy primary endpoint, studied efficacy of vaccinations, or were designed as a crossover trial were excluded, due to the potential for confounding variables that could disrupt an assessment of data analysis populations.

A PubMed search was used to identify relevant articles published in the nine selected journals. The search used the Medical Subject Heading (MeSH) term “anti-infective agents,” and was filtered to only yield RCTs published in the study timeframe. Search results (abstracts and articles, as necessary) were manually reviewed to exclude articles that were not RCTs of anti-infectives, as well as articles that met exclusion criteria.

Data from each article were extracted by two independently acting study investigators, with all investigators participating. Discrepancies were resolved by consensus under consultation with a third independent investigator. In order to build consistency among the four investigators, data collection was piloted for three articles that met study criteria, but were published prior to 2013. The following data were extracted in order to describe the sample of RCTs: journal of publication, intervention, medication class using the American Hospital Formulary Service (AHFS) classification of the intervention, indication for treatment, blinding, number of treatment arms, number of randomized patients, primary endpoint, and primary endpoint classification (i.e., continuous, ordinal, nominal). It was decided not to measure inter-rater reliability, since investigators extracted objective data only, without judging, interpreting, measuring, scoring, or rating its merits. Published recommendations suggest inter-rater reliability would be needed to assess consistency in the latter, but not the former, scenario [14].

Data gathered to address study objectives were collected in the same manner as described above. For each article, the primary data analysis approach (e.g., ITT, mITT, PP) described in the methods and used to present results was determined. It was identified during the data collection pilot that, on occasion, the primary data analysis approach in the methods differed from what was actually used in results, hence the decision to extract both data points. If clinical trial authors incorrectly classified an approach (e.g., stated that an article was ITT, when it had actually been modified in some way, such as requiring patients to receive at least one dose to be included in analysis), it was re-classified using the correct definition. Additionally, the total number and type(s) of data analysis approaches appearing in the article results were counted. Similarly, the primary approach used to handle missing data (e.g., LOCF) described in methods, and the number and type(s) of approaches to missing data present in the results were determined. Finally, each study was classified as a non-inferiority trial if non-inferiority methods were used to evaluate the primary endpoint, and as having favorable or unfavorable results according to whether the study was a superiority (i.e., improved efficacy compared to control) or non-inferiority (i.e., efficacy no worse than control) design.

Descriptive statistics using Microsoft Excel® were used to present article demographic information, as well as to address objectives 1, 2, and 3. Median and interquartile range were used to describe results for ordinal and non-parametric continuous data. Number and percentage were used to describe nominal data. The study was determined to be exempt from institutional review board (IRB) review upon consultation with a local IRB representative.

## Results

**Fig. 1:**
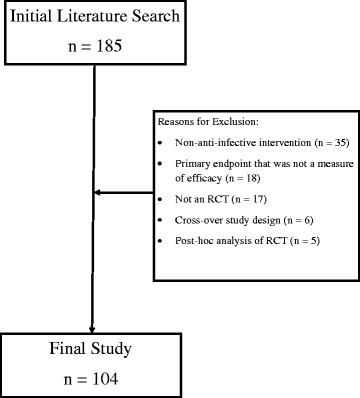
Articles Screened. Describes the number of articles screened in the initial literature search, as well as the reasons for article exclusion. RCT: Randomized, controlled trial

The initial literature search identified 185 articles, 81 (44 %) of which failed to satisfy study criteria (see Fig. 1). The most common reasons for removal were a non-anti-infective intervention (*n* = 35), having a primary endpoint that was not a measure of efficacy (*n* = 18), and not an RCT (*n* = 17).

The initial literature search identified 104 articles (56 %) that satisfied study criteria and were included in the analysis. See the Additional file 1 for citations of the final sample of studies. The most common medication classes represented were hepatitis C antivirals (*n* = 27, 26 %), antibacterials (*n* = 26, 25 %), and antiretrovirals (*n* = 22, 21 %). Most studies contained 2 (*n* = 64, 62 %) or 3 (*n* = 23, 22 %) treatment groups. Thirty studies (29 %) were non-inferiority trials; all others were superiority. Fifty percent (*n* = 52) of studies were double-blind and 46 % (*n* = 48) were open-label. Sixty-eight percent (*n* = 71) of studies used a nominal or dichotomous primary endpoint (e.g., clinical cure, microbiological cure, whether a patient met a target viral load), and 31 % (*n* = 32) used a continuous primary endpoint (e.g., mean viral load, time to event). Seventy-six percent (*n* = 79) had favorable results. See Table 2 for a full overview of descriptive results.

**Table 2:** Descriptive Information from Sample RCTs (*N* = 104)^a^

Medication class, no. (%)	Results
Hepatitis C antivirals	27 (26)
Antibacterials	26 (25)
Antiretrovirals	22 (21)
Antimalarials	6 (6)
Antifungals	4 (4)
Antimycobacterials	4 (4)
Miscellaneous antivirals	4 (4)
Neuraminidase inhibitors	4 (4)
Antihelminthics	2 (2)
Antiprotozoals	2 (2)
Nucleosides and nucleotides	2 (2)
Monoclonal antibodies	1 (1)
Treatment groups, no. (%)	
2	64 (62)
3	23 (22)
4	10 (10)
5	3 (3)
6	1 (1)
8	2 (2)
14	1 (1)
Study design, no. (%)	
Superiority	74 (71)
Non-inferiority	30 (29)
Enrolment, median (IQR)	395 (156 to 724)
Blinding, no. (%)	
Open-label	48 (46)
Single	1 (1)
Double	52 (50)
Not described	3 (3)
Endpoint type, no. (%)	
Nominal	71 (68)
Continuous	32 (31)
Unable to determine	1 (1)
Result type, no. (%)	
Favorable	79 (76)
Unfavorable	25 (24)

^a^ *IQR* Interquartile range

Of the included studies (*n* = 104), most studies described use of ITT or mITT as their primary approach to data analysis in their methods (*n* = 80, 77 %) and subsequently used one of these approaches as the primary data analysis approach in their results (*n* = 85, 82 %). Forty-one percent (*n* = 43) of studies used multiple data analysis approaches. No studies described use of data analysis approaches other than ITT, mITT, or PP. See Table 3 for a further overview of related results.

**Table 3:** Data Analysis Methods in Sample RCTs (*N* = 104)^a^

Primary approach described in methods, no. (%)	Results
mITT	42 (40)
ITT	38 (37)
Not stated or unclear	17 (16)
PP	4 (4)
Both mITT and PP	2 (2)
Both ITT and PP	1 (1)
Primary or secondary approach described in methods, no. (%)^b^	
mITT	53 (51)
ITT	53 (50)
PP	37 (35)
Primary approach used in results, no. (%)	
mITT	51 (49)
ITT	34 (33)
Not stated or unclear	10 (10)
PP	5 (5)
Both mITT and PP	2 (2)
Both ITT and PP	2 (2)

^a^ *ITT* intention-to-treat, *mITT* mITT, *PP* per protocol

^b^Not exclusive

For studies that used mITT as a primary or secondary approach to data analysis (*n* = 53), the most common modification was to assess all patients who received at least one dose of study drug (*n* = 24, 45 %). An additional 13 studies (25 %) included patients who received at least one dose of study drug and also satisfied one additional criterion (e.g., no resistance at baseline, present for at least one follow up, no violations of good clinical practice). Less common modifications included lack of microbiologically confirmed infection (*n* = 3), coinfection (*n* = 2), lack of primary outcome data (*n* = 2), and discontinuation for other reason (*n* = 1). Modifications were not indicated in 8 studies (15 %).

Of the 96 studies that used ITT, mITT, or both, most (*n* = 70, 73 %) did not describe practices used to handle missing data due to dropouts. One study did not indicate planned modifications, but did state that no patients had missing data. The described practices included assuming treatment failure (*n* = 13, 14 %), best observation carried forward (BOCF) (*n* = 3, 3 %), LOCF (*n* = 3, 3 %), excluding missing data from the analysis (*n* = 3, 3 %), and use of other single or multiple imputation methods (*n* = 3, 3 %).

Twenty-nine of the 30 non-inferiority trials (97 %) described use of ITT (*n* = 10, 33 %), mITT (*n* = 16, 53 %), or both (*n* = 3, 10 %) in the study methods. The remaining study described use of mITT in the study results, but not in the the study methods. However, only 15 (50 %) also described use of PP. Consistent with overall results, the approach used to account for missing data due to attrition was only described in 8 studies (27 %).

## Discussion

Results from this study strongly suggest that most RCTs of anti-infectives consistently employ ITT or mITT, which should provide the most conservative estimate of effect size (with the possible exception of non-inferiority trials). Few studies (4 %) described PP as their primary approach. This is in harmony with current best practices and recommendations for appropriate conduction of RCTs, recommending a multi-faceted approach to data analysis that centers on use of ITT [4]. This is also an improvement compared to previous evaluations of musculoskeletal and ear, nose, and throat (ENT) RCTs, where fewer studies (68 % and 12 %, respectively) used ITT or mITT as the primary data analysis population [6, 8]. While use of ITT is not specifically recommended in CONSORT, the relatively consistent use of ITT and mITT supports CONSORT Item 13, which advocate for clarity in describing the numbers of patients analyzed for each outcome, and providing reasons for patient exclusions [1].

Although the overall results regarding use of ITT in this sample of clinical trials were positive, a substantial minority of studies (17 %) failed to clearly define their primary population of analysis as recommended in CONSORT [1]. This failure decreases study transparency and puts the onus on the reader to determine whether an appropriate population was used when interpreting results. Misinterpretation of study group size may cause significant ramifications to study applicability, particularly in subspecialties such as infectious diseases where sample size might be lower than ideal. Additionally, considerable inconsistency between the primary population defined in methods and the one in used to illustrate results was anecdotally observed. These types of discrepancies can be particularly concerning when ITT is described in the methods, but less conservative mITT, or even PP, analyses are described in the study results. This could contribute to systematic overestimation of observed effect size of anti-infectives.

The most common modification used in mITT analyses required analyzed patients to receive at least one dose of medication (48 %). This minor modification would not be expected to greatly alter the observed effect size compared to ITT, and the finding is similar to a previous study where this was the most common modification (56 %) [15]. From a clinician’s perspective, a mITT population, depending on the modification, may provide a more representative picture of actual clinical practice. Practically though, increasing the number of modifications can introduce bias in the form of changing the overall study into a per-protocol analysis. Study authors and readers alike must balance these competing interests when designing and reading this type of manuscript. To the authors’ knowledge, there is no standard recommendation for the number or types of modifications considered appropriate for a mITT analysis. On a related note, it was observed that mITT was frequently miscategorized as ITT, which could be misleading to the reader depending on the degree of modification. This underscores previously reported findings that as few as 42 % of published RCTs claiming to use ITT actually assessed all randomized patients in their primary analysis [5].

A problem with ITT trials that is somewhat unique to infectious diseases research is the issue of how to account for patients who are randomized to a treatment group, but never positively have an established infection. Their inclusion into the study population while true to an ITT model provides increased noise, with limited benefit in terms of determining the overall effectiveness of an intervention. Simply excluding this population can also be viewed in a different light as a deviation from standard practice and less “real-world” in extrapolation. Our results suggest that microbiological confirmation of infectious is less commonly used as a mITT modification.

The overwhelming majority of RCTs that used ITT or mITT did not describe how investigators accounted for missing patient data due to missed visits or dropouts. These results were worse than prior studies where this information was provided in 58 to 65 % of RCTs [6, 16]. To the investigators’ knowledge, there is no current, rigorous standard regarding an ideal approach to this practice. The European Medicines Agency (EMA) has acknowledged lack of consensus in this area and suggests preferential use of more conservative approaches to handling missing data [17]. Some commonly used approaches to missing data (e.g., best observation carried forward [BCOF], LCOF) could easily result in a more “best case” result that might not truly reflect patient outcomes [4]. Other approaches, such as assuming treatment failure may be preferable as they would result in a more conservative estimation of effect size, especially in subspecialties such as infectious diseases, where dichotomous clinical endpoints (70 % in this study) are commonly employed [4, 17, 18]. Guidelines from EMA suggest assuming treatment failure may be most appropriate for clinical trials investigating response-type of endpoints, which comprised the majority in this study [17]. A positive finding was that the most commonly described approach was assuming treatment failure (13 %).

ITT or mITT was employed in the majority (97 %) of non-inferiority trials. However, it has been recommended that non-inferiority trials, and possibly all RCTs, should describe both ITT and PP results, and facilitate the reader’s focus on the more conservative findings [4, 13, 19–22]. Results from this study suggested that about half of non-inferiority trials of anti-infectives currently report both ITT and PP. This is another opportunity for improvement in transparency of study reporting that could yield more clinically applicable results.

Having focused on infectious disease medications, future studies could examine additional therapeutic classifications, such as cardiovascular medications. Additionally, subsequent work could focus on non-inferiority trials on a more global scale. A recent cross-sectional study of non-inferiority trials identified serious shortcomings in terms of reporting and justifying non-inferiority margins, and other information needed to interpret non-inferiority [23]. A broader assessment of non-inferiority trial reporting focused on data analysis populations could identify additional concerns. Along with a broader scope focusing on different populations, a more longitudinal approach spanning more years could also be constructed to evaluate not only the prevalence of the use of ITT over the years, but also how this modification to the effect size of studies changes with different approaches to RCTs.

Results from this study will be applicable for medical professionals in health systems, especially those with a role in antimicrobial stewardship or direct patient care. However, it should be cautioned that the consistent use of ITT in this sample may not be extrapolated to other subspecialties. Similarly, the proportion of non-inferiority trials used in infectious diseases is somewhat higher than expected in other disciplines, which further limits generalizability to other therapeutic areas [7, 24]. Another potential limitation is that the search purposefully focused on identifying and evaluating the most impactful infectious diseases RCTs published in highly regarded journals, and these results themselves may represent a “best case” scenario.

Results from this study highlight key information to consider when evaluating study methods and results, and a general lack of transparency in study reporting. Without a thorough understanding of the types and appropriateness of data being presented in a study, decisions about the use of a medication for a patient or its addition to the hospital formulary become dependent on the study authors’ presentation and conclusions. Lack of transparency could contribute to misinterpretation of important findings or use of an agent outside of its intended target population. Furthermore, the important shortcomings identified in this study will also be important for journal editors and peer reviewers to consider when evaluating RCTs, particularly those of anti-infectives, for publication.

## Conclusions

RCTs of anti-infectives commonly employ ITT and mITT populations for their primary analysis; however, a substantial portion of studies did not clearly report the population that was used. Most of these RCTs do not describe how missing data were addressed. Non-inferiority trials of anti-infectives do not consistently employ both ITT and PP populations.

## Additional file

Additional file 1: Sample of Randomized, Controlled Trials. Provides citations for the final sample of articles assessed in the project (*n* = 104). (DOCX 29 kb)

## Abbreviations

AHFS: American Hospital Formulary Service
BCOF: Best observation carried forward
CONSORT: The Consolidated Standards of Reporting Trials
EMA: European Medicines Agency
ENT: Ear, nose, and throat
IRB: Institutional review board
ITT: Intention-to-treat
LOCF: Last observation carried forward
MeSH: Medical Subject Heading
mITT: Modified intention-to-treat
PP: Per protocol
RCT: Randomized, controlled trial
