# Supplementary material for: Intention-to-treat and transparency of related practices in randomized, controlled trials of anti-infectives
Source: BMC Med Res Methodol. 2016 Aug 24;16(1):106. doi: 10.1186/s12874-016-0215-2 (PMC4997732; doi:10.1186/s12874-016-0215-2)
Supplement: Additional file 1: — Sample of Randomized, Controlled Trials. Provides citations for the final sample of articles assessed in the project (n = 104). (DOCX 29 kb) [file 12874_2016_215_MOESM1_ESM.docx]

ADDITIONAL FILE 1

Sample of Randomized, Controlled Trials (N = 104)

| 1. Afdhal N, Reddy KR, Nelson DR, Lawitz E, Gordon SC, Schiff E, et al. Ledipasvir and sofosbuvir for previously treated HCV genotype 1 infection. N Engl J Med. 2014;370:1483-93. |
| --- |
| 1. Afdhal N, Zeuzem S, Kwo P, Chojkier M, Gitlin N, Puoti M, et al. Ledipasvir and sofosbuvir for untreated HCV genotype 1 infection. N Engl J Med. 2014;370:1889-98. |
| 1. Altenburg J, deGraaff CS, Stienstra Y, Sloos JH, van Haren EH, Koppers RJ, et al. Effect of azithromycin maintenance treatment on infectious exacerbations among patients with non-cystic fibrosis bronchiectasis: the BAT randomized controlled trial. JAMA. 2013;309:1251-9. |
| 1. Arribas JR, Pialoux G, Gathe J, Di Perri G, Reynes J, Tebas P, et al. Simplification to coformulated elvitegravir, cobicistat, emtricitabine, and tenofovir versus continuation of ritonavir-boosted protease inhibitor with emtricitabine and tenofovir in adults with virologically suppressed HIV (STRATEGY-PI): 48 week results of a randomised, open-label, phase 3b, non-inferiority trial. Lancet Infect Dis. 2014;14:581-9. |
| 1. ARROW Trial team, Kekitiinwa A, Cook A, Nathoo K, Mugyenyi P, Nahirya-Ntege P, et al. Routine versus clinically driven laboratory monitoring and first-line antiretroviral therapy strategies in African children with HIV (ARROW). Lancet. 2013;381:1391-403. |
| 1. Awasthi S, Peto R, Read S, Richards SM, Pande V, Bundy D, et al. Population deworming every 6 months with albendazole in 1 million pre-school children in North India; DEVTA, a cluster-randomised trial. Lancet. 2013;381:1478-86. |
| 1. Benhamou Y, Moussalli J, Ratziu V, Lebray P, De Backer K, De Meyer S, et al. Telaprevir activity in treatment-naive patients infected hepatitis C virus genotype 4: a randomized trial. J Infect Dis. 2013;208:1000-7. |
| 1. Benjamin DK Jr, Hudak ML, Duara S, Randolph DA, Bidegain M, Mundakel GT, et al. Effect of fluconazole prophylaxis on candidiasis and mortality in premature infants: a randomized clinical trial. JAMA. 2014;311:1742-9. |
| 1. Beshir KB, Sutherland CJ, Sawa P, Drakeley CJ, Okell L, Mweresa CK, et al. Residual Plasmodium falciparum parasitemia in Kenyan children after artemisinin-combination therapy is associated with increased transmission to mosquitoes and parasite recurrence. J Infec Dis. 2013;208:2017-24. |
| 1. Blanken MO, Rovers MM, Molenaar JM, Winkler-Seinstra PL, Meijer A, Kimpen JL, et al. Respiratory syncytial virus and recurrent wheeze in healthy preterm infants. N Engl J Med. 2013;368:1791-9. |
| 1. Boucher HW, Wilcox M, Talbot GH, Puttagunta S, Das AF, Dunne MW. Once-weekly dalbavancin versus daily conventional therapy for skin infection. N Engl J Med. 2014;370:2169-79. |
| 1. Boulware DR, Meya DB, Muzoora C, Rolfes MA, Huppler Hullsiek K, et al. Timing of antiretroviral therapy after diagnosis of cryptococcal meningitis. N Engl J Med. 2014;370:2487-98. |
| 1. Boyd MA, Moore CL, Molina JM, Wood R, Madero JS, Wolff M, et al. Baseline HIV-1 resistance, virological outcomes, and emergent resistance in the SECOND-LINE trial: an exploratory analysis. Lancet HIV. 2015;2:42-51. |
| 1. Bwakura-Dangarembizi M, Kendall L, Bakeera-Kitaka S, Nahirya-Ntege P, Keishanyu R, Nathoo K, et al. A randomized trial of prolonged co-trimoxazole in HIV-infected children in Africa. N Engl J Med. 2014;370:41-53. |
| 1. Cahn P, Andrade-Villanueva J, Arribas JR, Gatell JM, Lama JR, Norton M, et al. Dual therapy with lopinavir and ritonavir plus lamivudine versus triple therapy with lopinavir and ritonavir plus two nucleoside reverse transcriptase inhibitors in antiretroviral-therapy-naive adults with HIV-1 infection: 48 week results of the randomised, open label, non-inferiority GARDEL trial. Lancet Infect Dis. 2014;14:572-80. |
| 1. Cahn P, Pozniak AL, Mingrone H, Shuldyakov A, Brites C, Andrade-Villanueva JF, et al. Dolutegravir versus raltegravir in antiretroviral-experienced, integrase-inhibitor-naive adults with HIV: week 48 results from the randomised, double-blind, non-inferiority SAILING study. Lancet. 2013;382:700-8. |
| 1. Chemaly RF, Ullmann AJ, Stoelben S, Richard MP, Bornhäuser M, Groth C, et al. Letermovir for cytomegalovirus prophylaxis in hematopoietic-cell transplantation. N Engl J Med. 2014;370:1781-9. |
| 1. Chen JS, Chan WK, Tsai KT, Hsu HH, Lin CY, Yuan A, et al. Simple aspiration and drainage and intrapleural minocycline pleurodesis versus simple aspiration and drainage for the initial treatment of primary spontaneous pneumothorax: an open-label, parallel-group, prospective, randomized, controlled trial. Lancet. 2013;381:1277-82. |
| 1. Chetchotisakd P, Chierakul W, Chaowagul W, Anunnatsiri S, Phimda K, Mootsikapun P, et al. Trimethoprim-sulfamethoxazole versus trimethoprim-sulfamethoxazole plus doxycycline as oral eradicative treatment for melioidosis (MERTH): a multicentre, double-blind, non-inferiority, randomised controlled trial. Lancet. 2014;383:807-14. |
| 1. Choopanya K, Martin M, Suntharasamai P, Sangkum U, Mock PA, Leethochawalit M, et al. Antiretroviral prophylaxis for HIV infection in injecting drug users in Bangkok, Thailand (the Bangkok Tenofovir Study): a randomised, double-blind, placebo-controlled phase 3 trial. Lancet. 2013;381:2083-90. |
| 1. Clotet B, Feinberg J, van Lunzen J, Khuong-Josses MA, Antinori A, Dumitru I, et al. Once-daily dolutegravir versus darunavir plus ritonavir in antiretroviral-naive adults with HIV-1 infection (FLAMINGO): 48 week results from the randomised open-label phase 3b study. Lancet. 2014;383:2222-31. |
| 1. Corey GR, Kabler H, Mehra P, Gupta S, Overcash JS, Porwal A, et al. Single-dose oritavancin in the treatment of acute bacterial skin infections. N Engl J Med. 2014;370:2180-90. |
| 1. Cotton MF, Violari A, Otwombe K, Panchia R, Dobbels E, Rabie H, et al. Early time-limited antiretroviral therapy versus deferred therapy in South African infants infected with HIV: results from the children with HIV early antiretroviral (CHER) randomised trial. Lancet. 2013;382:1555-63. |
| 1. Day JN, Chau TT, Wolbers M, Mai PP, Dung NT, Mai NH, et al. Combination antifungal therapy for cryptococcal meningitis. N Engl J Med. 2013;368:1291-302. |
| 1. DeVincenzo JP, Whitley RJ, Mackman RL, Scaglioni-Weinlich C, Harrison L, Farrell E, et al. Oral GS-5806 activity in a respiratory syncytial virus challenge study. N Engl J Med. 2014;371:711-22. |
| 1. Diacon AH, Pym A, Grobusch MP, de los Rios JM, Gotuzzo E, Vasilyeva I, et al. Multidrug-resistant tuberculosis and culture conversion with bedaquiline. N Engl J Med. 2014;371:723-32. |
| 1. Diener MK, Knebel P, Kieser M, Schüler P, Schiergens TS, Atanassov V, et al. Effectiveness of triclosan-coated PDS Plus versus uncoated PDS II sutures for prevention of surgical site infection after abdominal wall closure: the randomised controlled PROUD trial. Lancet. 2014;384:142-52. |
| 1. Durovni B, Saraceni V, Moulton LH, Pacheco AG, Cavalcante SC, King BS, et al. Effect of improved tuberculosis screening and isoniazid preventive therapy on incidence of tuberculosis and death in patients with HIV in clinics in Rio de Janeiro, Brazil: a stepped wedge, cluster-randomised trial. Lancet Infect Dis. 2013;13:852-8. |
| 1. ENCORE1 Study Group, Puls R, Amin J, Losso M, Phanuphak P, Nwizu C, et al. Efficacy of 400 mg efavirenz versus standard 600 mg dose in HIV-infected, antiretroviral-naïve adults (ENCORE1): a randomised, double-blind, placebo-controlled, non-inferiority trial. Lancet. 2014;383:1474-82. |
| 1. Eziefula AC, Bousema T, Yeung S, Kamya M, Owaraganise A, Gabagaya G, et al. Single dose primaquine for clearance of Plasmodium falciparum gametocytes in children with uncomplicated malaria in Uganda: a randomised, controlled, double-blind, dose-ranging trial. Lancet Infect Dis. 2014;14:130-9. |
| 1. Feld JJ, Kowdley KV, Coakley E, Sigal S, Nelson DR, Crawford D, et al. Treatment of HCV with ABT-450/r-ombitasvir and dasabuvir with ribavirin. N Engl J Med. 2014;370:1594-603. |
| 1. Ferenci P, Bernstein D, Lalezari J, Cohen D, Luo Y, Cooper C, et al. ABT-450/r-ombitasvir and dasabuvir with or without ribavirin for HCV. N Engl J Med. 2014;370:1983-92. |
| 1. Fry AM, Goswami D, Nahar K, Sharmin AT, Rahman M, Gubareva L, et al. Efficacy of oseltamivir treatment started within 5 days of symptom onset to reduce influenza illness duration and virus shedding in an urban setting in Bangladesh: a randomised placebo-controlled trial. Lancet Infec Dis. 2014;14:109-18. |
| 1. Gane EJ, Stedman CA, Hyland RH, Ding X, Svarovskaia WT, Symonds WT, et al. Nucleotide polymerase inhibitor sofosbuvir plus ribavirin for hepatitis C. N Engl J Med. 2013;1:34-44. |
| 1. Garcia HH, Gonzales I, Lescano AG, Bustos JA, Zimic M, Escalante D, et al. Efficacy of combined antiparasitic therapy with praziquantel and albendazole for neurocysticercosis: a double-blind, randomised controlled trial. Lancet Infect Dis. 2014;14:687-95. |
| 1. Giamarellos-Bourboulis EJ, Mylona V, Antonopoulou A, Tsangaris I, Koutelidakis I, Marioli A, et al. Effect of clarithromycin in patients with suspected Gram-negative sepsis: results of a randomized controlled trial. J Antimicrob Chemother. 2014;69:1111-8. |
| 1. Gillespie SH, Crook AM, McHugh TD, Mendel CM, Meredith SK, Murray SR, et al. Four-month moxifloxacin-based regimens for drug-sensitive tuberculosis. N Engl J Med. 2014;371:1577-87. |
| 1. Gilliams EA, Jumare J, Claassen CW, Thesing PC, Nyirenda OM, Dzinjalamala FK, et al. Chloroquine-azithromycin combination antimalarial treatment decreases risk of respiratory- and gastrointestinal-tract infections in Malawian children. J Infect Dis. 2014;210:585-92. |
| 1. Grinsztejn B, De Castro N, Arnold V, Veloso VG, Morgado M, Pilotto JH, et al. Raltegravir for the treatment of patients co-infected with HIV and tuberculosis (ANRS 12 180 Reflate TB): a multicentre, phase 2, non-comparative, open-label, randomised trial. Lancet Infect Dis. 2014;14:459-67. |
| 1. Grinsztejn B, Hosseinipour MC, Ribaudo HJ, Swindells S, Eron J, Chen YQ, et al. Effects of early versus delayed initiation of antiretroviral treatment on clinical outcomes of HIV-1 infection: results from the phase 3 HPTN 052 randomised controlled trial. Lancet Infect Dis. 2014;14:281-90. |
| 1. Haffizulla J, Hartman A, Hoppers M, Resnick H, Samudrala S, Ginocchio C, et al. Effect of nitazoxanide in adults and adolescents with acute uncomplicated influenza: a double-blind, randomised, placebo-controlled, phase 2b/3 trial. Lancet Infect Dis. 2014;14:609-18. |
| 1. Hamasuna R, Tanaka K, Hayami H, Yasuda M, Takahashi S, Kobayashi K, et al. Treatment of acute uncomplicated cystitis with faropenem for 3 days versus 7 days: multicentre, randomized, open-label, controlled trial. J Antimicrob Chemother. 2014;69:1675-80. |
| 1. Hilsden RJ, Macphail G, Grebely J, Conway B, Lee SS. Directly observed pegylated interferon plus self-administered ribavirin for the treatment of hepatitis C virus infection in people actively using drugs: a randomized controlled trial. Clin Infect Dis. 2013;2:90-6. |
| 1. Huang H, Li X, Zhu J, Ye S, Zhang H, Wang W, et al. Entecavir vs lamivudine for prevention of hepatitis B virus reactivation among patients with untreated diffuse large B-cell lymphoma receiving R-CHOP chemotherapy: a randomized clinical trial. JAMA. 2014;312:2521-30. |
| 1. Jacobson IM, Dore GJ, Foster GR, Fried MW, Radu M, Rafalsky VV, et al. Simeprevir with pegylated interferon alfa 2a plus ribavirin in treatment-naive patients with chronic hepatitis C virus genotype 1 infection (QUEST-1): a phase 3, randomised, double-blind, placebo-controlled trial. Lancet. 2014;384:403-13. |
| 1. Jacobson IM, Gordon SC, Kowdley KV, Yoshida EM, Rodriguez-Torres M, Sulkowski MS, et al. Sofosbuvir for hepatitis C genotype 2 or 3 in patients without treatment options. N Engl J Med. 2013;368:1867-77. (Fusion Trial) |
| 1. Jacobson IM, Gordon SC, Kowdley KV, Yoshida EM, Rodriguez-Torres M, Sulkowski MS, et al. Sofosbuvir for hepatitis C genotype 2 or 3 in patients without treatment options. N Engl J Med. 2013;368:1867-77. (Positron Trial) |
| 1. Janssen HL, Reesink HW, Lawitz EJ, Zeuzem S, Rodriguez-Torres M, et al. Treatment of HCV infection by targeting microRNA. N Engl J Med. 2013;368:1685-94. |
| 1. Jindani A, Harrison TS, Nunn AJ, Phillips PP, Churchyard GJ, Charalambous S, et al. High-dose rifapentine with moxifloxacin for pulmonary tuberculosis. N Engl J Med. 2014;371:1599-608. |
| 1. Knoll GA, Humar A, Fergusson D, Johnston O, House AA, Kim SJ, et al. Levofloxacin for BK virus prophylaxis following kidney transplantation: a randomized clinical trial. JAMA. 2014;312:2106-14. |
| 1. Kowdley KV, Gordon SC, Reddy KR, Rossaro L, Bernstein DE, Lawitz E, et al. Ledipasvir and sofosbuvir for 8 or 12 weeks for chronic HCV without cirrhosis. N Engl J Med. 2014;370:1879-88. |
| 1. Kowdley KV, Lawitz E, Crespo I, Hassanein T, Davis MN, DeMicco M, et al. Sofosbuvir with pegylated interferon alfa-2a and ribavirin for treatment-naïve patients with hepatitis C genotype-1 infection (ATOMIC): an open-label, randomized, multicentre phase 2 trial. Lancet. 2013;381:2100-7. |
| 1. Kowdley KV, Lawitz E, Poordad F, Cohen DE, Nelson DR, Zeuzem S, et al. Phase 2b trial of interferon-free therapy for hepatitis C virus genotype 1. N Engl J Med. 2014;370:222-32. |
| 1. Larrey D, Lohse AW, Trepo C, Bronowicki JP, Arastéh K, Bourlière M, et al. Antiviral effect, safety, and pharmacokinetics of five-day oral administration of Deleobuvir (BI 207127), an investigational hepatitis C virus RNA polymerase inhibitor, in patients with chronic hepatitis C. Antimicrob Agents Chemother. 2013;57:4727-35. |
| 1. Lawitz E, Mangia A, Wyles D, Rodriguez-Torres M, Hassanein T, Gordon SC, et al. Sofosbuvir for previously untreated chronic hepatitis C infection. N Engl J Med. 2013;368:1878-87. |
| 1. Lawitz E, Poordad FF, Pang PS, Hyland RH, Ding X, Mo H, et al. Sofosbuvir and ledipasvir fixed-dose combination with and without ribavirin in treatment-naive and previously treated patients with genotype 1 hepatitis C virus infection (LONESTAR): an open-label, randomised, phase 2 trial. Lancet. 2014;383:515-23. |
| 1. Lawitz E, Sulkowski MS, Ghalib R, Rodriguez-Torres M, Younossi ZM, et al. Simeprevir plus sofosbuvir, with or without ribavirin, to treat chronic infection with hepatitis C virus genotype 1 in non-responders to pegylated interferon and ribavirin and treatment-naive patients: the COSMOS randomised study. Lancet. 2014;384:1756-65. |
| 1. Lee HJ, Kim JI, Cheung DY, Kim TH, Jun EJ, Oh JH, et al. Eradication of Helicobacter pylori according to 23S ribosomal RNA point mutations associated with clarithromycin resistance. J Infect Dis. 2013;208:1123-30. |
| 1. Lee N, Hui DS, Zuo Z, Ngai KL, Lui GC, Wo SK, et al. A prospective intervention study on higher-dose oseltamivir treatment in adults hospitalized with influenza a and B infections. Clin Infect Dis. 2013;57:1511-9. |
| 1. Liou JM, Chen CC, Chem MJ, Chen CC, Chang CY, Fang YJ, et al. Sequential versus triple therapy for the first-line treatment of Helicobacter pylori: a multicenter, open-label, randomized trial. Lancet. 2013;381:205-13. |
| 1. Llanos-Cuentas A, Lacerda MV, Rueangweerayut R, Krudsood S, Gupta SK, Kochar SK, et al. Tafenoquine plus chloroquine for the treatment and relapse prevention of Plasmodium vivax malaria (DETECTIVE): a multicentre, double-blind, randomised, phase 2b dose-selection study. Lancet. 2014;383:1049-58. |
| 1. Llor C, Moragas A, Bayona C, Morros R, Pera H, Plana-Ripoll O, et al. Efficacy of anti-inflammatory or antibiotic treatment in patients with non-complicated acute bronchitis and discoloured sputum: randomised placebo controlled trial. BMJ. 2013;347:5762. |
| 1. Low JG, Sung C, Wijaya L, Wei Y, Rathore AP, Watanabe S, et al. Efficacy and safety of celgosivir in patients with dengue fever (CELADEN): a phase 1b, randomised, double-blind, placebo-controlled, proof-of-concept trial. Lancet Infect Dis. 2014;14:706-15. |
| 1. Manns M, Marcellin P, Poordad F, de Araujo ES, Buti M, Horsmans Y, et al. Simeprevir with pegylated interferon alfa 2a or 2b plus ribavirin in treatment-naive patients with chronic hepatitis C virus genotype 1 infection (QUEST-2): a randomised, double-blind, placebo-controlled phase 3 trial. Lancet. 2014;384:414-26. |
| 1. Manns M, Pol S, Jacobson IM, Marcellin P, Gordon SC, Peng CY, et al. All-oral daclatasvir plus asunaprevir for hepatitis C virus genotype 1b: a multinational, phase 3, multicohort study. Lancet. 2014;384:1597-605. |
| 1. Marty FM, Winston DJ, Rowley SD, Vance E, Papanicolaou GA, Mullane KM, et al. CMX001 to prevent cytomegalovirus disease in hematopoietic-cell transplantation. N Engl J Med. 2013;369:1227-36. |
| 1. Maude RJ, Silamut K, Plewes K, Charunwatthana P, Ho M, Abul Faiz M, et al. Randomized controlled trial of levamisole hydrochloride as adjunctive therapy in severe falciparum malaria with high parasitemia. J Infect Dis. 2014;209:120-9. |
| 1. Merle CS, Fielding K, Sow OB, Gninafon M, Lo MB, Mthiyane T, et al. A four-month gatifloxacin-containing regimen for treating tuberculosis. N Engl J Med. 2014;371:1588-98. |
| 1. Mfinanga SG, Kirenga BJ, Chanda DM, Mutayoba B, Mthiyane T, Yimer G, et al. Early versus delayed initiation of highly active antiretroviral therapy for HIV-positive adults with newly diagnosed pulmonary tuberculosis (TB-HAART): a prospective, international, randomised, placebo-controlled trial. Lancet Infect Dis. 2014;14:563-71. |
| 1. Molina I, Gómez i Prat J, Salvador F, Treviño B, Sulleiro E, et al. Randomized trial of posaconazole and benznidazole for chronic Chagas' disease. N Engl J Med. 2014;370:1899-908. |
| 1. Moran GJ, Fang E, Corey GR, Das AF, De Anda C, Prokocimer P. Tedizolid for 6 days versus linezolid for 10 days for acute bacterial skin and skin-structure infections (ESTABLISH-2): a randomised, double-blind, phase 3, non-inferiority trial. Lancet Infect Dis. 2014;14:696-705. |
| 1. Mujugira A, Magaret AS, Celum C, Baeten JM, Lingappa JR, Morrow RA, et al. Daily acyclovir to decrease herpes simplex virus type 2 (HSV-2) transmission from HSV-2/HIV-1 coinfected persons: a randomized controlled trial. J Infect Dis. 2013;208:1366-74. |
| 1. Oostdijk EA, Kesecioglu J, Schultz MJ, Visser CE, de Jonge E, van Essen EH, et al. Effects of decontamination of the oropharynx and intestinal tract on antibiotic resistance in ICUs: a randomized clinical trial. JAMA. 2014;312:1429-37. |
| 1. Osinusi A, Meissner EG, Lee YJ, Bon D, Heytens L, Nelson A, et al. Sofosbuvir and ribavirin for hepatitis C genotype 1 in patients with unfavorable treatment characteristics: a randomized clinical trial. JAMA. 2013;310:804-11. |
| 1. Ostrosky-Zeichner L, Shoham S, Vazquez J, Reboli A, Betts R, Barron MA, et al. MSG-01: A randomized, double-blind, placebo-controlled trial of caspofungin prophylaxis followed by preemptive therapy for invasive candidiasis in high-risk adults in the critical care setting. Clin Infec Dis. 2014;58:1219-26. |
| 1. [Pasaribu AP](http://www.ncbi.nlm.nih.gov/pubmed/?term=Pasaribu%20AP%5BAuthor%5D&cauthor=true&cauthor_uid=23926329), [Chokejindachai W](http://www.ncbi.nlm.nih.gov/pubmed/?term=Chokejindachai%20W%5BAuthor%5D&cauthor=true&cauthor_uid=23926329), [Sirivichayakul C](http://www.ncbi.nlm.nih.gov/pubmed/?term=Sirivichayakul%20C%5BAuthor%5D&cauthor=true&cauthor_uid=23926329), [Tanomsing N](http://www.ncbi.nlm.nih.gov/pubmed/?term=Tanomsing%20N%5BAuthor%5D&cauthor=true&cauthor_uid=23926329), [Chavez I](http://www.ncbi.nlm.nih.gov/pubmed/?term=Chavez%20I%5BAuthor%5D&cauthor=true&cauthor_uid=23926329), [Tjitra E](http://www.ncbi.nlm.nih.gov/pubmed/?term=Tjitra%20E%5BAuthor%5D&cauthor=true&cauthor_uid=23926329), et al. A randomized comparison of dihydroartemisinin-piperaquine and artesunate-amodiaquine combined with primaquine for radical treatment of vivax malaria in Sumatera, Indonesia. J Infect Dis. 2013;208:1906-13. |
| 1. Paton NI, Kityo C, Hoppe A, Reid A, Kambugu A, Lugemwa A, et al. Assessment of second-line antiretroviral regimens for HIV therapy in Africa. N Engl J Med. 2014;371:234-47. |
| 1. Poordad F, Hezode C, Trinh R, Kowdley KV, Zeuzem S, Agarwal K, et al. ABT-450/r-ombitasvir and dasabuvir with ribavirin for hepatitis C with cirrhosis. N Engl J Med. 2014;370:1973-82. |
| 1. Pozniak A, Markowitz M, Mills A, Stellbrink HJ, Antela A, Domingo P, et al. Switching to coformulated elvitegravir, cobicistat, emtricitabine, and tenofovir versus continuation of non-nucleoside reverse transcriptase inhibitor with emtricitabine and tenofovir in virologically suppressed adults with HIV (STRATEGY-NNRTI): 48 week results of a randomised, open-label, phase 3b non-inferiority trial. Lancet Infect Dis. 2014;14:590-9. |
| 1. Raffi F, Babiker AG, Richert L, Molina JM, George EC, Antinori A, et al. Ritonavir-boosted darunavir combined with raltegravir or tenofovir-emtricitabine in antiretroviral-naive adults infected with HIV-1: 96 week results from the NEAT001/ANRS143 randomised non-inferiority trial. Lancet. 2014;384:1942-51. |
| 1. Raffi F, Jaeger H, Quiros-Roldan E, Albrecht H, Belonosova E, Gatell JM, et al. Once-daily dolutegravir versus twice-daily raltegravir in antiretroviral-naive adults with HIV-1 infection (SPRING-2 study): 96 week results from a randomised, double-blind, non-inferiority trial. Lancet Infec Dis. 2013;13:927-35. |
| 1. Raffi F, Rachlis A, Stellbrink HJ, Hardy WD, Torti C, Orkin C, et al. Once-daily dolutegravir versus raltegravir in antiretroviral-naïve adults with HIV-1 infection: 48 week results from the randomized, double-blind, non-inferiority SPRING-2 study. Lancet. 2013;381:735-43. |
| 1. Rangaka MX, Wilkinson RJ, Boulle A, Glynn JR, Fielding K, van Cutsem G, et al. Isoniazid plus antiretroviral therapy to prevent tuberculosis: a randomised double-blind, placebo-controlled trial. Lancet. 2014;384:682-90. |
| 1. Ravis WR, Llanos-Cuentas A, Sosa N, Kreishman-Deitrick M, Kopydlowski KM, Nielsen C, et al. Pharmacokinetics and absorption of paromomycin and gentamicin from topical creams used to treat cutaneous leishmaniasis. Antimicrob Agents Chemother. 2013;57:4809-15. |
| 1. Regimbeau JM, Fuks D, Pautrat K, Mauvais F, Haccart V, Msika S, et al. Effect of postoperative antibiotic administration on postoperative infection following cholecystectomy for acute calculous cholecystitis: a randomized clinical trial. JAMA. 2014;312:145-54. |
| 1. RIVUR Trial Investigators, Hoberman A, Greenfield SP, Mattoo TK, Keren R, Mathews R, et al. Antimicrobial prophylaxis for children with vesicoureteral reflux. N Engl J Med. 2014;370:2367-76. |
| 1. Sedyaningsih ER, Malik MS, Setiawaty V, Trihono T, Burhan E, Aditama TY, et al. Effect of double dose oseltamivir on clinical and virological outcomes in children and adults admitted to hospital with severe influenza: double blind randomised controlled trial. BMJ. 2013;346:3039. |
| 1. Serisier DJ, Martin ML, McGuckin MA, Lourie R, Chen AC, Brain B, et al. Effect of long-term, low-dose erythromycin on pulmonary exacerbations among patients with non-cystic fibrosis bronchiectasis: the BLESS randomized controlled trial. JAMA. 2013;309:1260-7. |
| 1. Solomkin JS, Ramesh MK, Cesnauskas G, Novikovs N, Stefanova P, Sutcliffe JA, et al. Phase 2, randomized, double-blind study of the efficacy and safety of two dose regimens of eravacycline versus ertapenem for adult community-acquired complicated intra-abdominal infections. Antimicrob Agents Chemother. 2014;58:1847-54. |
| 1. SPARTAC Trial Investigators, Fidler S, Porter K, Ewings F, Frater J, Ramjee G, et al. Short-course antiretroviral therapy in primary HIV infection. N Engl J Med. 2013;368:207-17. |
| 1. Speich B, Ame SM, Ali SM, Alles R, Huwyler J, Hattendorf J, et al. Oxantel pamoate-albendazole for Trichuris trichiura infection. N Engl J Med. 2014;370:610-20. |
| 1. Sulkowski M, Pol S, Mallolas J, Fainboim H, Cooper C, Slim J, et al. Boceprevir versus placebo with pegylated interferon alfa-2b and ribavirin for treatment of hepatitis C virus genotype 1 in patients with HIV: a randomised, double-blind, controlled phase 2 trial. Lancet Infect Dis. 2013;13:597-605. |
| 1. Sulkowski MS, Gardiner DF, Rodriguez-Torres M, Reddy KR, Hassanein T, et al. Daclatasvir plus sofosbuvir for previously treated or untreated chronic HCV infection. N Engl J Med. 2014;370:211-21. |
| 1. Sulkowski MS, Kang M, Matining R, Wyles D, Johnson VA, Morse GD, et al. Safety and antiviral activity of the HCV entry inhibitor ITX5061 in treatment-naive HCV-infected adults: a randomized, double-blind, phase 1b study. J Infect Dis. 2014;209:658-67. |
| 1. Thomas KS, Crook AM, Nunn AJ, Foster KA, Mason JM, Chalmers JR, et al. Penicillin to prevent recurrent leg cellulitis. N Engl J Med. 2013:368:1695-703 |
| 1. Trehan I, Goldbach HS, LaGrone LN, Meuli GJ, Wang RJ, Maleta KM, et al. Antibiotics as part of the management of severe acute malnutrition. N Engl J Med. 2013;368:425-35. |
| 1. Tubiana R, Mandelbrot L, Le Chenadec J, Delmas S, Rouzioux C, Hirt D, et al. Lopinavir/ritonavir monotherapy as a nucleoside analogue-sparing strategy to prevent HIV-1 mother-to-child transmission: the ANRS 135 PRIMEVA phase 2/3 randomized trial. Clin Infect Dis. 2013;57:891-902. |
| 1. van Dongen TM, van der Heijden GJ, Venekamp RP, Rovers MM, Schilder AG. A trial of treatment for acute otorrhea in children with tympanostomy tubes. N Engl J Med. 2014;370:723-33. |
| 1. Wald A, Corey L, Timmler B, Magaret A, Warren T, Tyring S, et al. Helicase-primase inhibitor pritelivir for HSV-2 infection. N Engl J Med. 2014;370:201-10. |
| 1. Walmsley SL, Antela A, Clumeck N, Duiculescu D, Eberhard A, Gutiérrez F, et al. Dolutegravir plus abacavir-lamivudine for the treatment of HIV-1 infection. N Engl J Med. 2013;369:1807-18. |
| 1. Yi TJ, Walmsley S, Szadkowski L, Raboud J, Rajwans N, Shannon B, et al. A randomized controlled pilot trial of valacyclovir for attenuating inflammation and immune activation in HIV/herpes simplex virus 2-coinfected adults on suppressive antiretroviral therapy. Clin Infect Dis. 2013;57:1331-8. |
| 1. Zanger P, Nurjadi D, Gabor J, Gaile M, Kremsner PG. Effectiveness of rifaximin in prevention of diarrhoea in individuals travelling to south and southeast Asia: a randomised, double-blind, placebo-controlled, phase 3 trial. Lancet Infec Dis. 2013;13:946-54. |
| 1. Zeuzem S, Jacobson IM, Baykal T, Marinho RT, Poordad F, Bourlière M, et al. Retreatment of HCV with ABT-450/r-ombitasvir and dasabuvir with ribavirin. N Engl J Med. 2014;370:1604-14. |
| 1. Zeuzem S, Soriano V, Asselah T, Bronowicki JP, Lohse AW, Müllhaupt B, et al. Faldaprevir and deleobuvir for HCV genotype 1 infection. N Engl J Med. 2013;369:630-9. |
